# Dataset on antitumor properties of silver nanoparticles from *Gloriosa superba* (L.) seed on Dalton Lymphoma Ascites (DLA) tumor: Facile and biocompatible approach

**DOI:** 10.1016/j.dib.2017.08.003

**Published:** 2017-08-03

**Authors:** Muthukirshnan Saradhadevi, Murugesan Gnanadesigan, Gnanajothi Kapildev, Dhakshinamoorthy Vasanth

**Affiliations:** a Department of Biochemistry, Bharathiar University, Coimbatore 641046, Tamil Nadu, India; b Department of Microbial Biotechnology, Bharathiar University, Coimbatore 64106, Tamil Nadu, India

**Keywords:** Silver nanoparticles, Green synthesis, MTT, Trypan blue, Flow cytometry, *Gloriosa superba*, Antitumor properties

## Abstract

The dataset depicted in this article related to our earlier article entitled “Phytofabrication and encapsulated of silver nanoparticles from *Gloriosa Superba*” (Saradha Devi et al., 2017) [1], which reports the characteristic features (UV Visible spectra, FTIR, SEM, TEM, DLS, Zeta potential and XRD analysis) of the *Gloriosa superba* biosynthesised silver nanoparticles (AgNPs). In this context, the present dataset was provided to identify the antioxidant, antitumor and apoptotic (in DLA cells) properties with the synthesized AgNPs. The result enlightens the AgNPs exhibits antitumor, apoptotic activity in DLA cells and antioxidant properties. The results of the *in vivo* experiments increased life span of liver cells in DLA induced tumour mice and not showed any histopathological variations between control and DLA induced mice animals. The HPTLC examination of the *Gloriosa superba* (L.) seed extract infers the presence of colchicines derivatives as a major alkaloid sources.

**Specifications Table**

**Table:** 

Subject area	*Biology*
More specific subject area	*Nanomedicine, Cancer*
Type of data	*Figures*
How data was acquired	*UV- Vis* Spectrophotometer, Light Microscope, Flow cytometry and HPTLC analysis,
Data format	*Plotted and analyzed*
Experimental features	*in vitro antioxidant assays with UV- Vis Spectrophotometer.*
	*in vitro antitumor assay with MTT proliferation assay, trypan blue exclusion assay and Apoptotic measurement activity (Flow cytometer analysis).*
	*in vivo cytotoxic studies with Swiss albino mice transplanted with DLA tumor cells.*
	*HPTLC performed with Ethyl acetate-Methanol-Water 10:1.35:1mobile phases and captured the images at Visible light, UV 254 nm and UV366* *nm.*
Data source location	11° 12′ 51.6528′′ N and 79° 21′ 40.5612′′ E.
Data accessibility	Data provided as graph and accessible within article

**Value of data**

• The HPTLC data displays the colchicine at 254 nm (peak 9) and the 13 unknown peaks in seed extract (Fig. 1b–e).

**Fig. 1:**
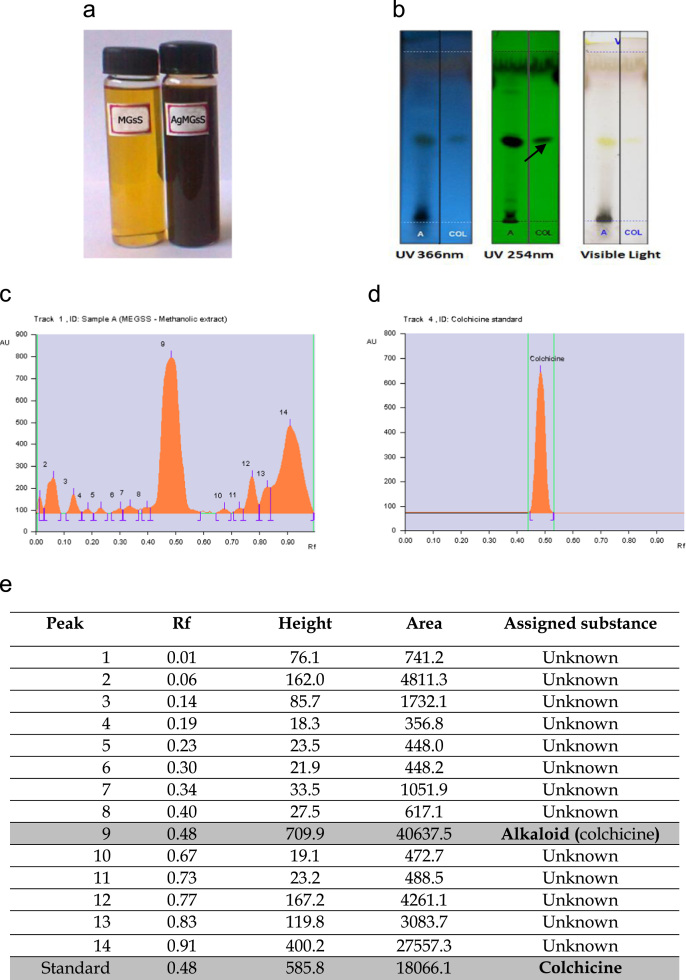
(a) Methanolic extract synthesised silver nanoparticles; (b) HPTLC Chromatogram pattern of methanolic extract at 366 nm, 254 nm and visible light (The arrow indicates the presence of colchicines in the methanolic extract); (c) HPTLC densitogram of the methanol extract with eleven distinct peaks; (d) HPTLC densitogram of the standard marker colchicine; (e): HPTLC peak table of the metahnolic extract.
• The antioxidant property of the seed extract and AgNPs are comparable with the vitamin C (Fig. 2).

**Fig. 2:**
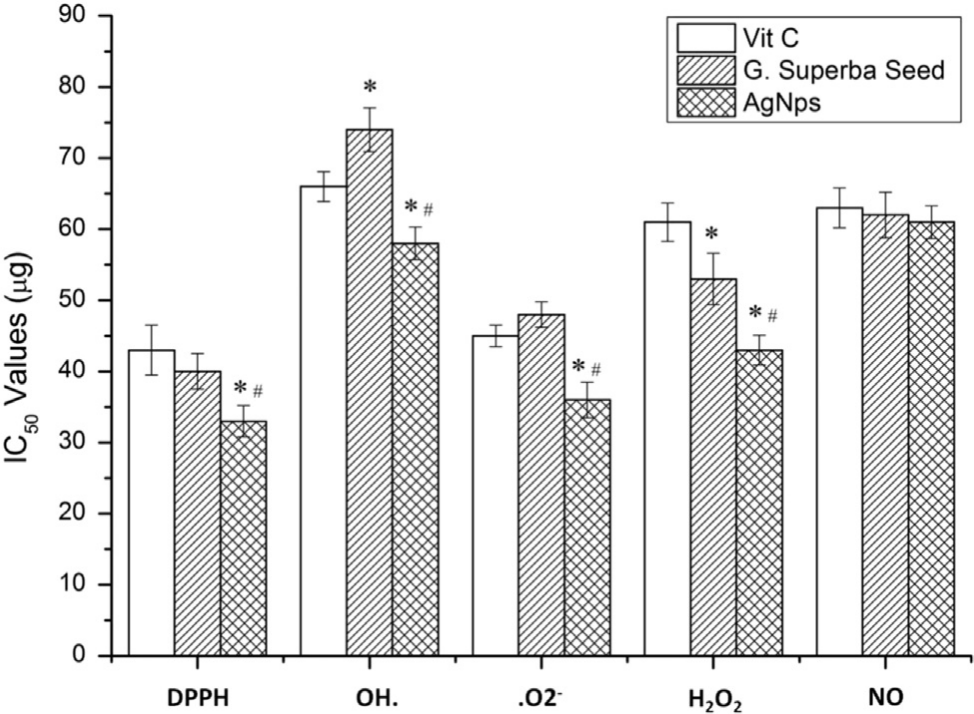
Free radical scavenging assay of *G. superba* seed methanolic extract and synthesized AgNPs against DPPH, Hydroxyl, Superoxide, Hydrogen Peroxide and Nitric oxide. *p<0.05 compared to Vitamin C; ^#^*p*<0.05 compared to *G. Superba* Seed (One way ANOVA followed by Tukey's multiple comparison test).
• The flow cytometry data analysis confirms the apoptotic nature of the seed extract and AgNPs to DLA cells (Fig. 3a and b)

**Fig. 3:**
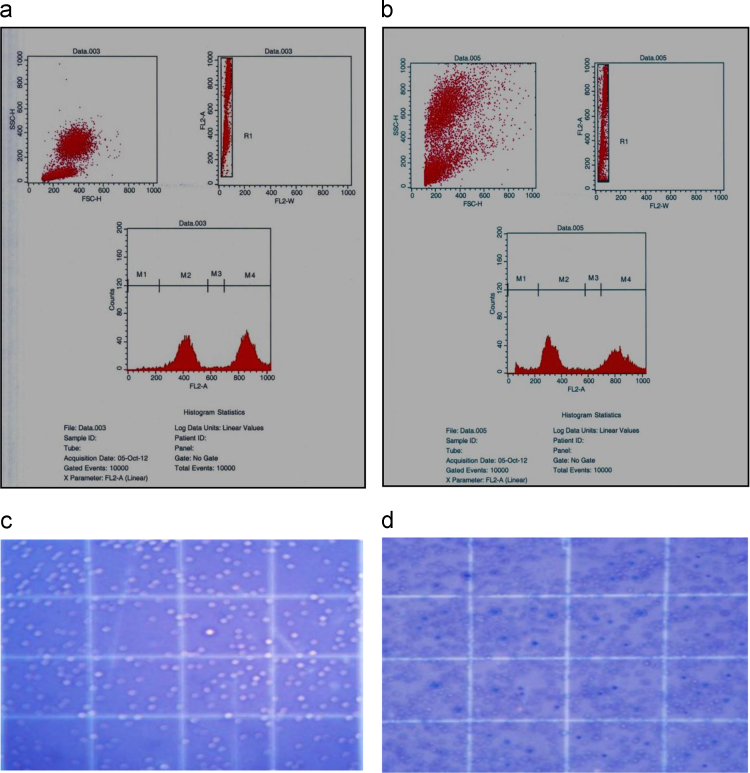
Apoptotic effect of AgNPs to DLA cells by fluorescent-activated cell sorting analysis (a) DLA cells with no apoptotic effect by fluorescent-activated cell sorting analysis; (b) Apoptotic effect of AgNPs to DLA cells by fluorescent-activated cell sorting analysis; c) DLA cell lines (Live cells appear as white round cell); (d) AgNPs treated DLA cell lines (Dead cells appear as in blue colour cells).
• The MTT assay data shows the toxicity to the DLA cell lines with IC_50_ values of 29 µg and 21 µg for seed extract and for AgNPs respectively.
• The data analysis of the trypan blue assay exhibited the concentration dependent cytotoxic effect in DLA tumor cells with the seed extract and AgNPs (Fig. 3c and d).

## 1 Data

**Fig. 4:**
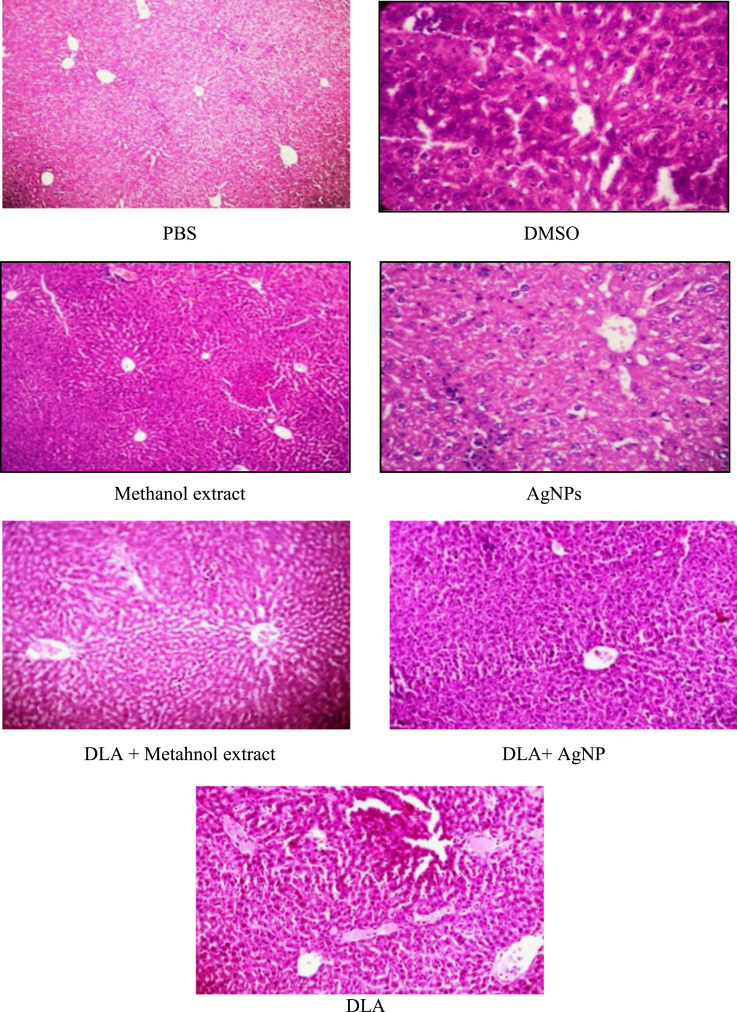
Histological view of the hepatocytes of controls, methanol extract, and AgNPs treated with normal and DLA induced Swiss albino mice.

The dataset depicted in this article related to our earlier article entitled “Phytofabrication and encapsulated of silver nanoparticles from *Gloriosa superba”* [1]. The free radicle scavenging properties of the *G. superba* seed extract and AgNPs were represented by DPPH, hydroxyl radicle, superoxide radicle, hydrogen peroxide and nitric oxide assays. The free radical scavenging efficacy of AgNPs was found to be higher than that of *G. superba* seed extract [2]. The toxicity effect of the seed extract and AgNPs on DLA tumor cells was carried out with MTT assay [3], [4]. The data analysis of the *in vitro* anticancer property of *G. superba* seed extract and AgNPs were carried out with the DLA cell lines. The *in vivo* antitumor data study shows the average life span of DLA tumor bearing mice identified as 20 days, but the seed extract and AgNPs administrated animals were increased the life span up to 72 and 75 days respectively (data not shown) [5]. The histopathological data of liver section of treated and controls groups showed normal lobular architecture with intact central vein and sinusoids and normal portal triad architectures (Fig. 4).

## 2 Materials and Methods

### 2.1 Preparation of the extract and synthesis of silver nanoparticles

*Gloriosa superba* seeds were collected from outskirts of Thanjavur district, Tamil Nadu province in India and authenticated. The 10 g of seeds powder was extracted with 150 ml of methanol. Then 10 ml of methanol seed extract was mixed with 90 ml of 1 mM silver nitrate solution and incubated for a period of 15 h at room temperature. The color changed from yellowish brown to dark brown color indication the formation of silver nanoparticles (Fig. 1a).

### 2.2 in vitro antioxidant activity

Antioxidant analysis of methanol extract of *G. superba* seed and AgNPs was evaluated by assessing their scavenging of DPPH, hydroxyl radical (OH.), Superoxide radical (.O2)− radicals and non-radicals such as Hydrogen peroxide (H_2_O_2_) and Nitric oxide (NO) against the standard Vitamin C by standard protocols [6].

### 2.3 Antitumor activity

#### 2.3.1 Maintenance of animals

Swiss albino mice of 5–7 weeks old (20–25 g) were bought from Animal breeding station, Kerala Agricultural University, Thrissur, India. The mice were maintained under dark/light cycle (14/10 h). The mice were acclimatized to laboratory conditions for 15 days before the commencement of the experiments. All procedures described were reviewed and approved by the University Animals Ethical Committee (Reg no: 623/02/b/CPCSEA).

#### 2.3.2 in vitro antitumor activity

*in vitro* antitumor activity of methanol extract of *G. superba* seed and AgNPs was evaluated by assessing their cytotoxic effect to DLA tumor cells by MTT proliferation assay, Trypan blue exclusion assay and Apoptotic measurement activity [7].

#### 2.3.3 in vivo antitumor activity

*in vivo* cytotoxic studies were carried out using the ED_50_ of methanol extract of *G. superba* seed and AgNPs to follow the antitumor activity in terms of increase in life Span and histological examination of seven groups (Groups A to G) of (6 mice/group) Swiss albino mice transplanted with DLA tumor cells [8].

Grouping of animals.

The Swiss albino mice were divided into 7 groups with six animals in each treatment groups as follows:

Group A: Mice received 100 μl of PBS and served as a control;.

Group B: Mice received 100 μl of DMSO and served as vehicle control.

Group C: Mice received methanolic extract (33 μg in 100 μl (ED_50_) of DMSO).

Group D: Mice received AgNPs (25 μg in 100 μl of DMSO).

Group E: Mice received one acute dose of 1×10^6^ DLA tumour cells in 100 µl of PBS on the first day of the experimental period and also ED_50_ dose of methanolic extract, throughout the experimental tenure.

Group F: Mice received one acute dose of 1×10^6^ DLA tumour cells in 100 µl of PBS on the first day of the experimental period and also ED_50_ dose of AgNPs, throughout the experimental tenure.

In Group G: Mice, tumour was induced by the administration of one acute dose of 1× 10^6^ DLA tumour cells in 100 µl of PBS on the first day of the experimental period.

The experiments were carried out for 20 days and 60 days. At the end of the study the mice were sacrificed after an overnight fasting. The liver was dissected, blotted of blood and washed with PBS of pH 7.2. The histological examination of the liver of all the experimental mice was carried out.

### 2.4 High performance thin layer liquid chromatography

The seed extract of *G. superba* was dissolved with HPLC grade methanol 100 mg/0.5 ml. The samples loaded plate was kept in TLC twin trough developing chamber with respective mobile phase (Ethyl acetate-Methanol-Water 10:1.35:1) up to 90 mm and captured the images at Visible light, UV 254 nm and UV366 nm [9].

## References

[1] Devi M. Saradha, Ashokkumar K., Annapoorani S. (2017). Phytofabrication and encapsulated of silver nanoparticles from *Gloriosa Superba*. Mater. Lett..

[2] Mani A., Lakshmi S.S., Gopal V. (2012). Bio-mimetic synthesis of silver nanoparticles and evaluation of its free radical scavenging activity. Int. J. Biol. Pharm. Res..

[3] Subramanian V., Suja S. (2012). Green synthesis of silver nanoparticles using *Coleus amboinicus lour, antioxitant activity* and in vitro cytotoxicity against Ehrlich’s Ascite carcinoma. J. Pharm. Res..

[4] Sivabalan M., Shering A., Reddy P., Vasudevaiah V., Jose A., Nigila G. (2011). Formulation and evaluation of 5-fluorouracil loaded chitosan and Eudragit nanoparticles for cancer therapy. Pharm. Glob., (IJCP).

[5] Jayaseelan R.S., Vijayan F.P., Mathesvaran M., Sureshm V., Padikkala J. (2012). Cytotoxic and antitumor activity of methonolic extracts *Desmodium triangulare* (Retz) merr. Root. Int. J. Pharm. Pharm. Sci..

[6] Ruch R.J., Cheng S.J., Klaunig J.E. (1989). Prevention of cytotoxicity and inhibition of intracellular communication by antioxidant catechins isolated from Chinese green tea. J. Carcinog..

[7] Salomi M.J., Panikkar K.R. (1989). Cytotoxic action of Nigella sativa seeds. Amala Res. Bull..

[8] Chaves G.M., Auxiliadora de Q.M., Maria dos A.A., Leao C., Lopes L.S. (2004). Model of experimental infection in healthy and immunosuppressed Swiss albino mice (*Mus musculus*) using *Candida albicans* strains with different patterns of enzymatic activity. Braz. J. Microbiol..

[9] Puri A., Ahmad A., Panda B.P. (2010). Development of an HPTLC-based diagnostic method for invasive aspergillosis. Biomed. Chromatogr..

